# A TNF-NF-κB-STAT3 loop triggers resistance of glioma-stem-like cells to Smac mimetics while sensitizing to EZH2 inhibitors

**DOI:** 10.1038/s41419-019-1505-5

**Published:** 2019-03-19

**Authors:** Bakhos A. Tannous, Christian E. Badr

**Affiliations:** 1 Department of Neurology, Massachusetts General Hospital, Boston, MA USA; 2 Neuroscience Program, Harvard Medical School, Boston, MA USA; 3 Experimental Therapeutics and Molecular Imaging Laboratory, Massachusetts General Hospital, Building 149, 13th Street, Charlestown, MA 02129 USA

Glioblastomas (GBM), the most common malignant primary brain tumors, remain incurable with high mortality and poor survival of less than 14 months^1^. Effective treatment modalities are lacking due to the location of these tumors complicating drug delivery, their invasive nature decreasing the possibility of complete tumor resection, as well as their heterogeneity with several genetic aberrations that drive tumor growth. Deciphering the molecular mechanisms promoting therapeutic resistance or enhancing the ability of tumor initiation glioma-stem-like cells (GSCs) to proliferate and drive tumor growth is key to identifying effective therapies. Because of their inherent plasticity, GSCs can adapt to therapeutic insults and respond by activating various transcription factors that promote survival. For instance, the transcription factor nuclear factor-kappa B (NF-κB), potently activated by inflammatory cytokines such as TNFα and IL-6, promotes invasion, self-renewal and proliferation, and therapeutic resistance in GSCs^2,3^.

**Fig. 1:**
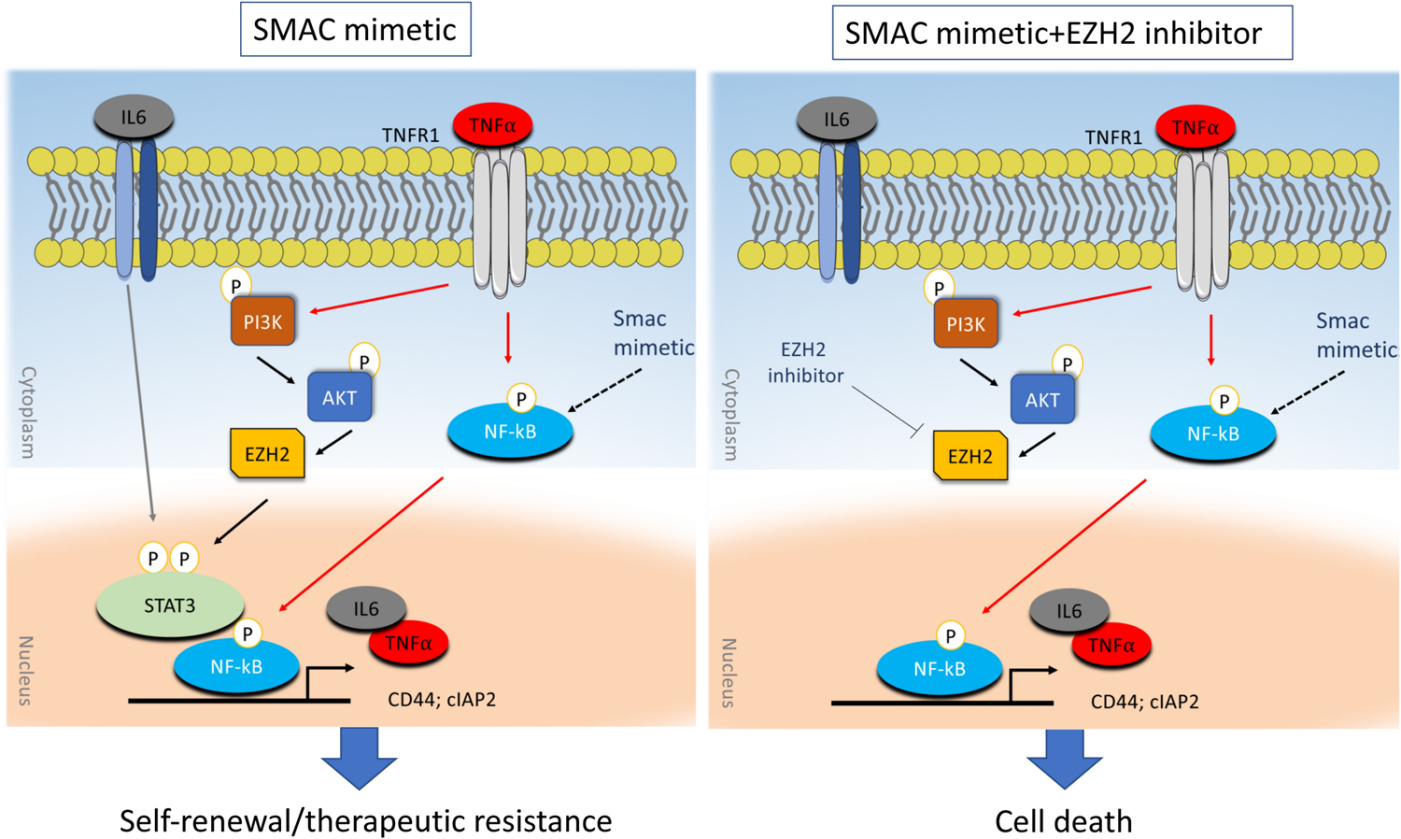
Schematic representation of activated signaling cascades in GSCs following treatment with SM; NF-kB activation promotes an autocrine/paracrine TNFα/IL6 signaling which in turn activates AKT/EZH2 and STAT3, consequently promoting self-renewal and therapeutic resistance. On the other hand, the combination of SM with EZH2 inhibitors results in cell death and GSCs depletion

Inhibitor of apoptosis proteins (IAPs) represent  a family of functionally/structurally related proteins that prevent apoptotic cell death and thus contribute to therapeutic resistance^4^. Several members of the IAPs family including cellular IAP2 (cIAP2) are upregulated in GBM and are associated with poor disease outcome. Therefore, counteracting IAP-mediated therapeutic resistance, using IAP antagonists commonly known as SMAC mimetics (SM), represents an appealing therapeutic strategy^5^. Recent studies have renewed the interest in SM for GBM therapy due to their demonstrated role as potent adjuvants to immunotherapy^6^. SM primarily induce proteasomal degradation of IAPs, consequently activating NF-κB signaling and promoting TNFα-mediated cell death^7^. TNFα is a pleiotropic cytokine that could induce cytotoxic cell death but could also trigger cell survival, proliferation, and invasion. The latter outcome is commonly observed in inherently resistant cancer cells, particularly in cancer stem cells. Our recently published work^8^ sought to examine the molecular response of GSCs to SM. Treatment with two SM of different chemical structure failed to cause significant long-term cytotoxicity in GSCs. Unexpectedly, the SM birinapant increased neurospheres formation and GSCs migration. Additionally, GSCs that were expanded over several weeks in the presence of birinapant showed superior resistance to radiation therapy in vivo. Overall, these findings suggest that treatment with SM stimulates self-renewal and enhances resistance in GSCs. We observed that the treatment of GSCs with birinapant promoted a sustained and prolonged activation of NF-κB, driven by TNFα and IL6. These cytokines create an autocrine/paracrine stimulation leading to a sustained NF-κB activity, aberrant activation of STAT3 signaling, and increased expression of pro-oncogenic proteins known to promote a cancer stem cell phenotype such as CD44 (Fig. 1). The cross-talk between NF-κB and STAT3 drives tumor progression and promotes cancer stemness in multiple malignancies including gliomas^9,10^. Further analysis of transcriptional targets of NF-κB and STAT3 revealed an increased cIAP2 expression mediated by TNFα upon treatment with SM. This observation was particularly interesting since these compounds target IAPs for degradation and cIAP2 upregulation confers resistance to SM^11^.

SM are likely more effective as adjuvant therapeutics in combination with cytotoxic agents in order to enhance their therapeutic potential. Given that constitutive activation of NF-κB and/or STAT3 enhances GSCs' resistance to SM-induced cell death, leading to increased expression of anti-apoptotic regulators such as Bcl-2, Bcl-xL, Mcl-1, and cIAP2, and promoting therapeutic resistance, it is tempting to combine NF-κB/STAT3 inhibitors with SM. Direct inhibition of NF-κB and to a lesser extent STAT3 could potentially lead to undesired side effects caused by immunosuppression and compromised immune response. Additionally, specific targeting of STAT3 has yet to be clinically validated. As an alternative strategy, based on a previously reported mechanism of EZH2-mediated activation of STAT3 in GSCs^12^, we sought to test the combination of EZH2 inhibitors with SM. The combination of small molecules inhibitors of EZH2 at subtoxic doses with SM resulted in a dramatic decrease in GSCs viability, suggesting a novel combination strategy for GBM. EZH2 inhibition also increased cytotoxicity of GSCs treated with recombinant TNFα. Thus, combination of EZH2 inhibitors and SM (or other therapeutics that activate NF-κB/TNFα) could be clinically relevant since both compounds are currently undergoing clinical evaluation for different malignancies. Given the implication of TNFα, NF-κB, and STAT3 in mesenchymal transition, typically associated with poor prognosis and resistance, and the role of EZH2 is sustaining STAT3 activation, it is likely that EZH2 activation facilitates a mesenchymal switch. This is indeed supported by previous evidence showing EZH2 to promote mesenchymal transition in cancer^13,14^. In order to evaluate the biological and clinical implication of these findings, testing EZH2 inhibitors and SM combination in preclinical GBM models, and a complete understanding of molecular pathways modulated by such treatment, is warranted. While clinical trials evaluating the efficacy of SM as antineoplastic agents are ongoing, understanding potential resistance mechanisms and designing rationale-based combination therapies are critical for improving clinical outcomes, and could provide novel therapeutic strategies for highly aggressive malignancies such as GBM.
